# A New Tool to Quantify Receptor Recruitment to Cell Contact Sites during Host-Pathogen Interaction

**DOI:** 10.1371/journal.pcbi.1003639

**Published:** 2014-05-29

**Authors:** Matthew S. Graus, Carolyn Pehlke, Michael J. Wester, Lisa B. Davidson, Stanly L. Steinberg, Aaron K. Neumann

**Affiliations:** 1 Department of Pathology, University of New Mexico, Albuquerque, New Mexico, United States of America; 2 Center for Spatiotemporal Modeling of Cell Signaling, University of New Mexico, Albuquerque, New Mexico, United States of America; 3 Center for Spatiotemporal Modeling of Cell Signaling and Department of Mathematics and Statistics, University of New Mexico, Albuquerque, New Mexico, United States of America; 4 Department of Pathology, University of New Mexico, Albuquerque, New Mexico, United States of America; 5 Center for Spatiotemporal Modeling of Cell Signaling and Department of Mathematics and Statistics, University of New Mexico, Albuquerque, New Mexico, United States of America; 6 Department of Pathology, University of New Mexico, Albuquerque, New Mexico, United States of America; Johns Hopkins University, United States of America

## Abstract

To understand the process of innate immune fungal recognition, we developed computational tools for the rigorous quantification and comparison of receptor recruitment and distribution at cell-cell contact sites. We used these tools to quantify pattern recognition receptor spatiotemporal distributions in contacts between primary human dendritic cells and the fungal pathogens *C. albicans*, *C. parapsilosis* and the environmental yeast *S. cerevisiae*, imaged using 3D multichannel laser scanning confocal microscopy. The detailed quantitative analysis of contact sites shows that, despite considerable biochemical similarity in the composition and structure of these species' cell walls, the receptor spatiotemporal distribution in host-microbe contact sites varies significantly between these yeasts. Our findings suggest a model where innate immune cells discriminate fungal microorganisms based on differential mobilization and coordination of receptor networks. Our analysis methods are also broadly applicable to a range of cell-cell interactions central to many biological problems.

## Author Summary

Specialized cell-cell contacts are a common theme in cell biology. These structures increase sensitivity and specificity of cellular activation and information flow in contexts ranging from activation of immune responses to transmission of nerve action potentials. *Candida* species fungal pathogens are responsible for significant morbidity associated with mucocutaneous infections as well as mortality () caused by bloodstream infections. The initial contact between innate immune cells and *Candida* results in a cell-cell contact between host and microbe. Leukocytes mobilize a network of receptors to these contact sites, and these receptors collaborate to recognize molecular patterns characteristic of microbial surfaces. Receptor recruitment, activation, and cross-talk are critical determinants of the evolution of signaling that directs the activation of downstream immune responses. However, host-pathogen contacts with fungi are complex and variable, and accurate quantification of receptor distribution in space and time is difficult with existing image analysis tools. Therefore, we have developed computational algorithms and a user interface that allows the scientist to both visualize and quantify receptor distribution in and recruitment to cell-cell contacts. We have used this software to show significant differences in contact site receptor accumulation and organization for three different host-fungal contact sites with environmental and pathogenic fungi. We also explored the correlation of contact site characteristics with the important functional outcome of phagocytosis.

## Introduction

*C. albicans* is a commensal of the human oropharyngeal cavity, gastrointestinal tract and female lower reproductive tract. It is also a significant opportunistic pathogen [1]. Infection by *Candida* species causes illnesses ranging from superficial mucosal infections that markedly diminish quality of life to bloodstream infections associated with high mortality. Systemic fungal infections by *C. albicans* have emerged as important causes of sickness and death in immunocompromised patients [2]. Some major risk factors associated with Candidemia involve neutropenia and prolonged hospitalization (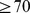 days) involving in-dwelling medical devices which can become infected with *Candida* [3]. There is 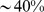 mortality rate associated with systemic *Candida* infection and an increased incidence of these types of infections in cancer patients [4]–[6]. For instance, *Candida* accounts for about one quarter of the fungal infections seen in leukemia patients [7]. During tissue colonization and invasion, *C. albicans* can undergo a transition from ellipsoidal yeast to filamentous hyphae, and this dimorphism is thought to be important for the infectious process. *C. parapsilosis* is one of the more commonly isolated non- *albicans Candida* species and is particularly problematic in neonates. It is clinically identified in 7–21% of systemic Candidiasis cases, where it is associated with 10–28% mortality [8]–[10]. *C. parapsilosis* colonizes human skin and nails, which is significant for its role in nosocomial infection [11]. *C. parapsilosis* can also be isolated from non-human animals, soil and physical surfaces [12]. *S. cerevisiae* is an environmental yeast most commonly associated with baking and fermentation processes. It is an exceedingly rare human pathogen, but can infect severely immune compromised patients [13]. The differing lifestyles of the three species compared may require different adhesive properties and regulation of cell wall structures so these fungi may adapt to and persist within their various niches. Nevertheless, they all contain grossly similar cell wall polysaccharide components and organization.

Around 85% of the *C. albicans* cell wall is made up of diverse carbohydrates—primarily mannoproteins, 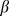-glucans, and chitin [14]–[16]. Chitin is deposited at sites deep within the cell wall and also exhibits some surface-accessibility at yeast bud scars [17]–[19]. However, the outermost layer of the *Candida* cell wall presents an external surface dominated by N-linked glycans which are comprised mostly of mannans [20] with punctate exposure of 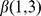
- and 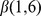-glucans [17]–[19]. The cell wall contains a variety of mannosylated species including protein N- and O-linked 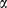-mannosides [16], *β*-linked mannosides within N-linked mannan [21] and phospholipomannan [22], [23]. Cell wall polysaccharides are essentially immobile on the time scale of host-pathogen interaction. *Candida* may modulate the degree of ligand exposure during infection [24].

Because the fungal cell wall is so complex, leukocytes must use multiple receptors in order to detect, interact with and initiate immune responses to fungal pathogens [20], [25], [26]. Innate immune cells, such as dendritic cells (DCs), rely on pattern recognition receptors (PRRs) to identify fungal pathogens. These PRRs recognize pathogen-associated molecular patterns, which are characteristic molecular signatures of microbial biology [1], [27], [28]. Significant PRRs for fungal mannan recognition include the C-type lectins (CTLs) DC-SIGN, CD206 (Mannose Receptor), Dectin-2 and Mincle (N-linked mannan); the Toll-like receptors TLR4 (O-linked mannan) and TLR2 (phospholipomannan); and Galectin-3 (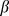-linked mannosides) [23], [25], [26], [29], [30]. 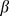-glucans are also immunogenic ligands of Dectin-1 (a CTL) and can be recognized by the 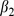 integrin Mac-1 [31]. These receptors are expected to be relatively mobile in the plasma membrane.

Recent research advances have clarified the identities of many receptors involved in fungal recognition, and increasingly (i.e., for DC-SIGN and Dectin-1), signal transduction cascades have been elucidated [32]. For *Candida albicans*, there is evidence that receptors can tailor specific downstream signaling and cytokine responses depending on the morphological state of the pathogen. For example, investigators have reported that CLR-mediated recognition of both *C. albicans* yeasts and hyphae [33], [34] and *C. parapsilosis* [35] results in divergent T helper cell polarization responses. Nevertheless, the specific contributions of individual receptors and their integration into the larger, multi-receptor system of fungal pattern recognition is not clear. Despite their ability to bind important pathogenic antigens, genetic ablation of CD206 or a murine homolog of DC-SIGN, SIGNR1, has been shown to have little impact on host defense in murine models of Candidiasis and *S. mansoni* infection [36], [37]. However, the existence of redundant systems for mannan sensing and species-specific differences in CTL function likely explain these findings. Furthermore, the interaction of *Candida* mannan with CD206 and DC-SIGN is well recognized as an important event in the generation of cytokine responses and phagocytosis by leukocytes [25], [32], [38]–[40]. While the functional consequences of CTL engagement are partially overlapping, evidence suggests that specific CTLs may be important for specific functions such as pathogen binding, phagocytosis and inflammatory cytokine generation [41] and co-engagement can modify CTL function [42].

Innate immune antigen presenting cells, such as dendritic cells, are some of the first responders to fungal infections and they also activate adaptive immune responses that are critical for clearing *Candida* infections [43], [44]. The earliest event that occurs in response to a *Candida* infection is the formation of a contact between an innate immune cell and the pathogenic fungal cell, which then determines the course of downstream signaling to activate inflammatory responses. Understanding the biology of fungal recognition requires elucidation of 1) the transport of C-type Lectins and other pattern recognition receptors to the site of host-microbe interaction, 2) rearrangement and coalescence of these receptors to achieve lateral segregation or clustering, and 3) the initiation of signaling cascades at the host-microbe contact site.

**Figure 1:**
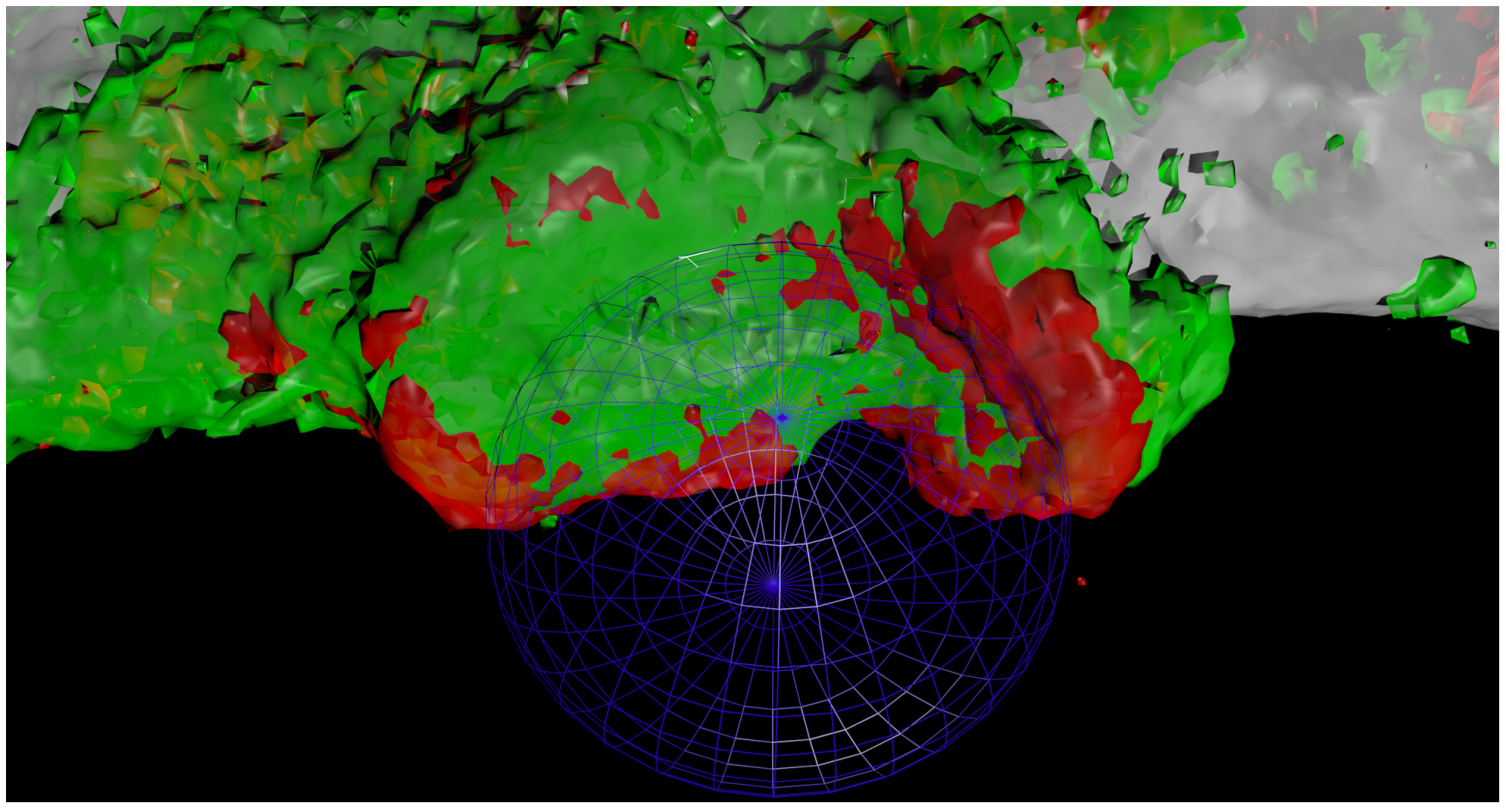
Human immature dendritic cells (DCs) form complex three dimensional host-pathogen contacts between DC plasma membrane (gray) and *Candida albicans* yeast (position depicted by blue wireframe sphere). Fungal recognition involves the deployment of C-type lectins such as DC-SIGN (green) and CD206 (red) to these contact sites. These contact sites have typical dimensions that match the diameter of *C. albicans* yeast, in the range of 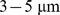 diameter.

Despite the identification of various receptors involved in fungal recognition, many questions remain regarding the mechanisms of receptor assembly at host-fungal pathogen contact sites, the role of receptor aggregation at nano- and micrometer length scales [45], and the spatiotemporal regulation of receptor cross-talk [31], [46]. Key to answering these questions are tools that provide rigorous quantification of receptor redistribution and signaling at host pathogen contacts. The distribution of CTLs can be imaged at high resolution by three-dimensional multicolor confocal laser scanning microscopy (3D CLSM). A major difficulty in developing analysis tools is that the imaging data is collected using rectangular voxels while the yeast cell is nearly spherical and rigid, so the contact between the yeast and dendritic cell is part of an essentially spherical surface (Fig. 1).

To overcome this difficulty, we developed geometric algorithms that construct spherical voxels that contain the yeast cell. The intensities in the rectangular voxels are transferred to the spherical voxels and then projected onto the surface of a sphere that approximates the surface of the yeast cell using weighted sums along the radial direction. The approximation is lenient, so a spectrum of geometries of the contact site are tolerable as long as they reside on a roughly spherical surface or within a spherical shell. We used these tools to quantitatively compare the differences in the contact site organization for the pathogens *C. albicans*, *C. parapsilosis*, and the environmental yeast *S. cerevisiae*. Some previous studies have used spherical coordinates to analyze biological data in ways that are related to, but significantly extended by, what we do here [47]–[50]. For instance, the tool we describe solves the above problems with particular attention to accurate transfer of intensity information to spherical voxels, use of equal area surface pixels for orientation-independence of contact site quantification, and a user-friendly interface that automatically computes a variety of spatial statistical measurements to assist in analysis of cell-cell contacts.

## Results

### Host-Microbe Contact Site Formation and Labeling

**Figure 2:**
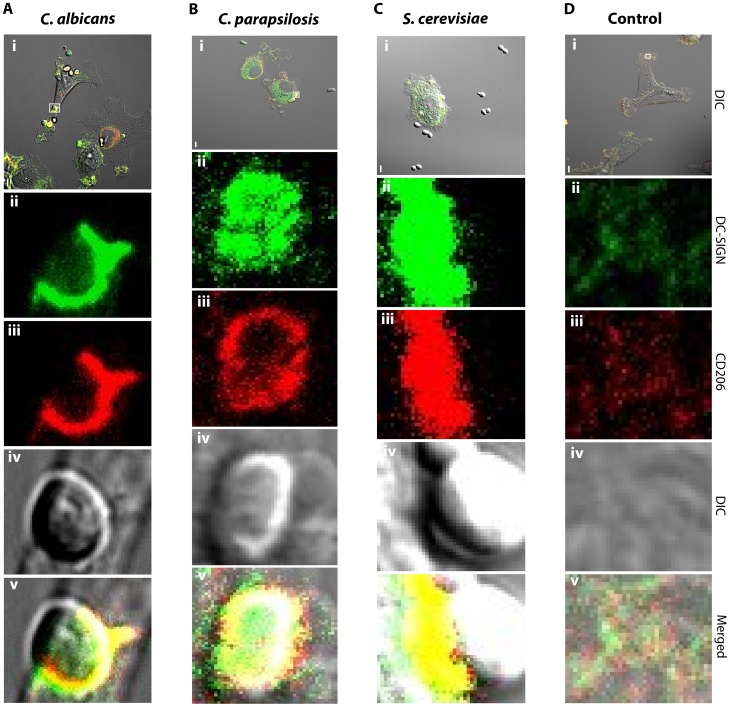
Contact site distribution of DC-SIGN and CD206. A) A dendritic cell contact site with *C. albicans*, depicting the distribution of two receptors, DC-SIGN and CD206, with confocal laser scanning fluorescence microscopy. A single Z-plane from the 3D image stack shows receptor intensity and distributions in an example contact site. Panels represent low magnification DIC (i; scale bar = 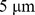) and contact site details of DC-SIGN (ii), CD206 (iii), DIC (iv) and merged fluorescence channels (v). B–D) Similar imaging and analysis performed for contacts with *C. parapsilosis* (B), *S. cerevisiae* (C) or resting dendritic cell control membranes (D).

We cultured immature DCs with yeast cells for various times, then fixed the cells and fluorescently labeled the CTLs, DC-SIGN and CD206, as well as the DC membrane lipids, as described in Materials and Methods. We used one environmental yeast (*Saccharomyces cerevisiae*) and two pathogenic yeasts (*Candida albicans* and *Candida parapsilosis*) to form the host-microbe contacts. We have chosen to focus our attention on these fungi because *Saccharomyces* and *Candida* cell wall composition and structure are thought to be mostly similar (see Discussion), yet the innate immune system is often called upon to discriminate between harmless environmental fungi and pathogenic ones. Furthermore, we have focused on two receptors prominently involved in mannan recognition in order to elucidate how mannan sensing is orchestrated. Three color 3D fluorescence distributions at cell-pathogen contact sites were measured by 3D CLSM. Representative examples of the initial data are shown in Fig. 2.

We compared DC-SIGN and CD206 at fungal contacts formed in response to *S. cerevisiae*, *C. albicans* and *C. parapsilosis* with respect to spatiotemporal patterns of receptor entry at 0, 1 and 4 hours of exposure to yeasts. These time points were chosen to focus on stable contact sites. Previous research has shown that the majority of zymosan particles bound to human DCs exhibit stable extracellular contacts over hours, and CTL signaling can occur from extracellular contacts with fungal ligands and from internal compartments over prolonged periods of time [51]–[53].

### Receptor Intensity Distribution Patterns

**Figure 3:**
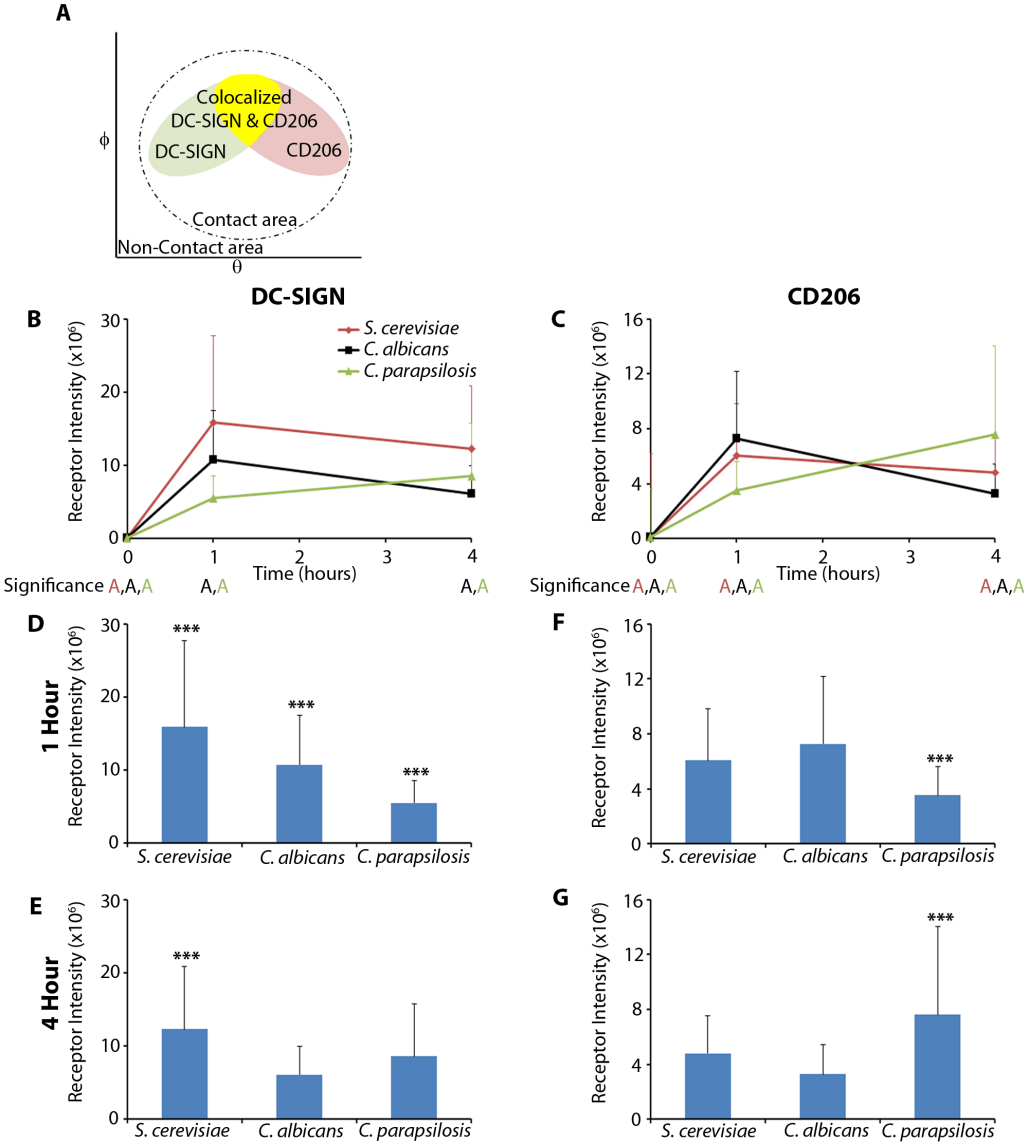
Receptor intensity distribution patterns. A) Schematic representation of a model host-microbe contact site representing spatial distributions of DC-SIGN and CD206 intensity on the curved contact site membrane as a function of their spherical coordinates (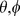) relative to the yeast center. The dashed line depicts the boundary of the contact site as defined by DC plasma membrane stain present at the fungal cell wall edge. Within the contact area, there are regions of receptor colocalization and single receptor localization. B,C) Comparison of receptor total intensity over the entire contact for (B) DC-SIGN and (C) CD206 at times post exposure to yeasts (0, 1, 4 hours). D,E) Statistical comparison of total receptor intensity over the entire contact site for DC-SIGN at (D) one hour and (E) four hours post exposure. F,G) Statistical comparison of total receptor intensity over the entire contact site for CD206 at (F) one hour and (G) four hours post exposure. Statistical significance was determined by ANOVA, Tukey post-hoc test (*** and “A”: 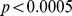; letter colors matched to figure legend) with 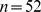 samples per donor for 1 and 4 hours and 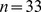 samples per donor for 0 h. Comparisons are between the designated point and other time points of the same color (B,C) or between the different species of yeast (D,E,F,G). Values and error bars displayed in all panels are presented as means and standard deviations.

We observed differential CTL spatiotemporal distribution patterns in contact sites with the three fungal species. These contact sites contained zones that were colocalized (on a diffraction limited scale) or single positive (schematically represented in Fig. 3A). *S. cerevisiae* and *C. albicans* provoked the greatest amount of DC-SIGN and CD206 recruitment respectively, within the first hour, and then both lost receptor intensity in the fourth hour. In contrast, *C. parapsilosis* continued to recruit significant amounts of both receptors from the start of the experiment into the fourth hour (Fig. 3B,C). The slower recruitment of DC-SIGN by *C. parapsilosis* resulted in contact site accumulations that were 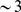 times less than *S. cerevisiae* and 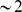 times less than *C. albicans* at the first hour (Fig. 3D). However, by the fourth hour, *C. parapsilosis* had recruited 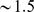 times more DC-SIGN than *C. albicans* and was still significantly less than *S. cerevisiae* (Fig. 3E). Similarly, *C. parapsilosis* recruited CD206 slowly, 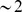 times less than both the other yeasts (Fig. 3F), but by the fourth hour recruited 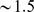 times more than *S. cerevisiae* and 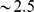 times more than *C. albicans* (Fig. 3G). We observed large increases in DC-SIGN intensity recruited to the contact site in the first hour for *S. cerevisiae*, *C. albicans* and *C. parapsilosis* : 161-fold, 140-fold and 82-fold, respectively. Likewise, we observed contact site enrichments, albeit lower in magnitude, for CD206 intensity in the first hour for *S. cerevisiae*, *C. albicans* and *C. parapsilosis* : 63-fold, 73-fold and 34-fold, respectively. This data suggested that DC-SIGN and CD206 recruitment patterns varied in a manner that was quite sensitive to the species of yeast being recognized by the DC—both in terms of the amount and spatiotemporal distribution of receptor recruited. It was further notable that DC-SIGN, and CD206 to a somewhat lesser extent, was highly enriched in contact sites relative to resting cells and that both CTLs were well recruited to *C. albicans* contacts, as seen for the other yeasts as well.

### Receptor Area Distribution Patterns

Receptor total intensity increase might derive from an increase in contact site area and/or increase of receptor density in contact sites. We proceeded to examine the contribution of these factors, starting with an assessment of contact site area. For all cases, we found that augmentation of CTL contact site area occurred most dramatically in the first hour, which is expected based on previously reported findings with macrophages interacting with *C. albicans* [54].

**Figure 4:**
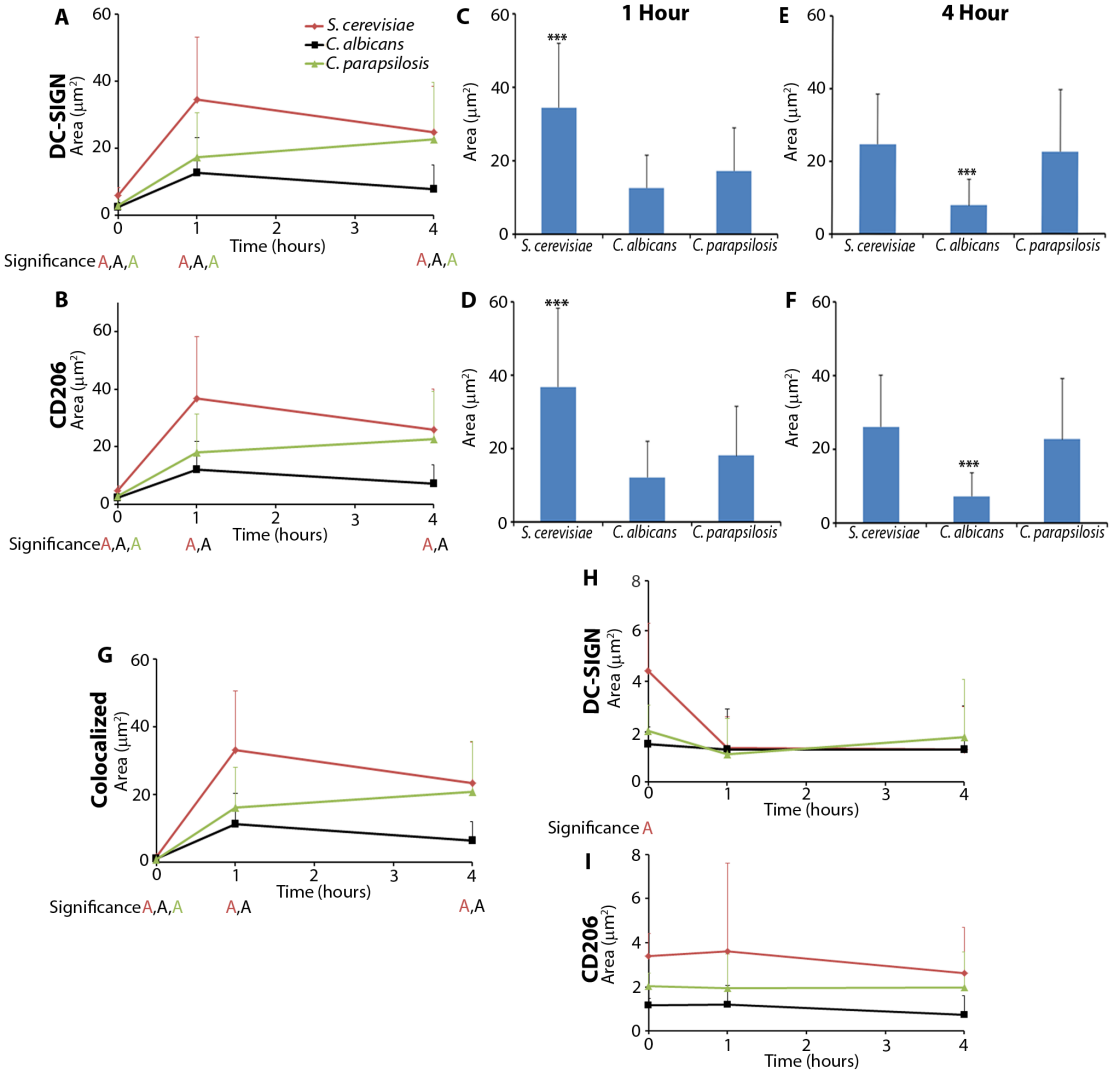
Receptor area distribution patterns. A,B) Comparison of changes in total area, as a fraction of the entire contact as defined by the plasma membrane, for (A) DC-SIGN and (B) CD206 at time points post exposure to yeast (0, 1, 4 hours). C,D) Statistical comparison of total receptor percent area over the entire contact site for (C) DC-SIGN and (D) CD206 at one hour post exposure. E,F) Statistical comparison of total receptor percent area over the entire contact site for (E) DC-SIGN and (F) CD206 at four hours post exposure. G,H,I) Comparison of area distribution patterns by change in area over the entire contact site for (G) Colocalized receptors, (H) single positive DC-SIGN, and (I) single positive CD206 at time points post exposure to yeast (0, 1, 4 hours). Statistical significance was determined by ANOVA, Tukey post-hoc test (*** and “A”: 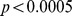; letter colors match to figure legend) with 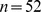 samples per donor for 1 and 4 hours and 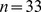 samples per donor for 0 hour. Comparisons are between the designated point and other time points of the same color (A,B,G,H,I) or between the different species of yeast (C,D,E,F). Values and error bars displayed in all panels are presented as means and standard deviations.

We found significant differences in the evolution of contact site area for DC-SIGN and CD206 amongst the three fungal species used to challenge DCs. *S. cerevisiae* was notable for the fact that it produced the contacts with largest area occupied by either receptor over the course of the experiment (Fig. 4A,B). In contrast, both *C. albicans* and *C. parapsilosis* contacts were significantly smaller at one hour for both individual CTL contact site areas and total contact area (Fig. 4A,B). *S. cerevisiae* contacts contained at least 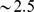 and 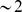 times larger DC-SIGN and CD206 area than either of the other yeasts at one hour (Fig. 4C,D), and 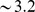 times greater DC-SIGN and CD206 area relative to *C. albicans* at four hours (Fig. 4E,F). *S. cerevisiae* contacts rapidly and effectively expanded, likely indicating a strong cytoskeletal response driving pseudopod extension for engulfment of the yeast. In contrast, *C. albicans* failed to produce contact site areas comparable to *S. cerevisiae* at either time point (Fig. 4A,B). This may reflect a blunted cytoskeletal response to *C. albicans* and poorer engulfment, which is addressed further below. While *S. cerevisiae* and *C. albicans* contacts were quantitatively different but followed a similar pattern of CTL spatiotemporal distribution, *C. parapsilosis* contacts were qualitatively different from the other yeasts contacts in that they exhibited a slow, progressive area increase (Fig. 4A,B). This progressive area increase for *C. parapsilosis* mirrored a similar trend seen for receptor recruitment (Fig. 3B,C).

To address the question of whether contact site area correlated with fungal particle size, we measured the major and minor radii of *S. cerevisiae*, *C. albicans* and *C. parapsilosis* yeasts (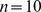 each) from DIC images (data not shown). From these measurements we also calculated mid-sectional elliptical perimeters. Upon comparing these results by ANOVA and post-hoc test, we determined that *C. albicans* and *S. cerevisiae* yeast sizes were not significantly different for any of these quantities. *C. parapsilosis* did exhibit significantly larger major radii (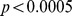) and elliptical perimeters (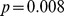) compared to *S. cerevisiae*. *S. cerevisiae* generated the largest contact sites and *C. albicans* had the smallest contacts, yet these yeasts were similar in size. Therefore, we conclude that contact site size is not dictated by particle size but is more likely a reflection of the DCs response to the particle.

Next we wanted to examine what population of the CTLs contributed to the increase in area. As illustrated in Fig. 3A, the contact can be divided into membrane regions with receptors that are colocalized at the limit of resolution (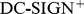, 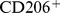) and single positive (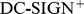, 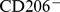; or 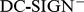, 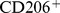) regions. After analyzing the different populations of CTLs within the contact site, we found that the significant increase in total receptor area was primarily due to an increase in colocalized populations of CTLs in the contact site (Fig. 4G). On the contrary, both populations of single-positive CTLs (DC-SIGN and CD206) did not change significantly throughout the experiment and comprised a small fraction of the total contact site (Fig. 4H,I).

Taken together, our observations demonstrate that the spatial assembly of the contact site structure is regulated differentially in response to the fungal species presented. It is also clear that all examined contact sites prominently featured increased predominance of receptor-colocalized membrane areas. Notably, *C. albicans* recognition by DCs generated the smallest contact sites despite our finding that this yeast was not deficient in recruiting DC-SIGN or CD206 total intensity.

### Receptor Density

**Figure 5:**
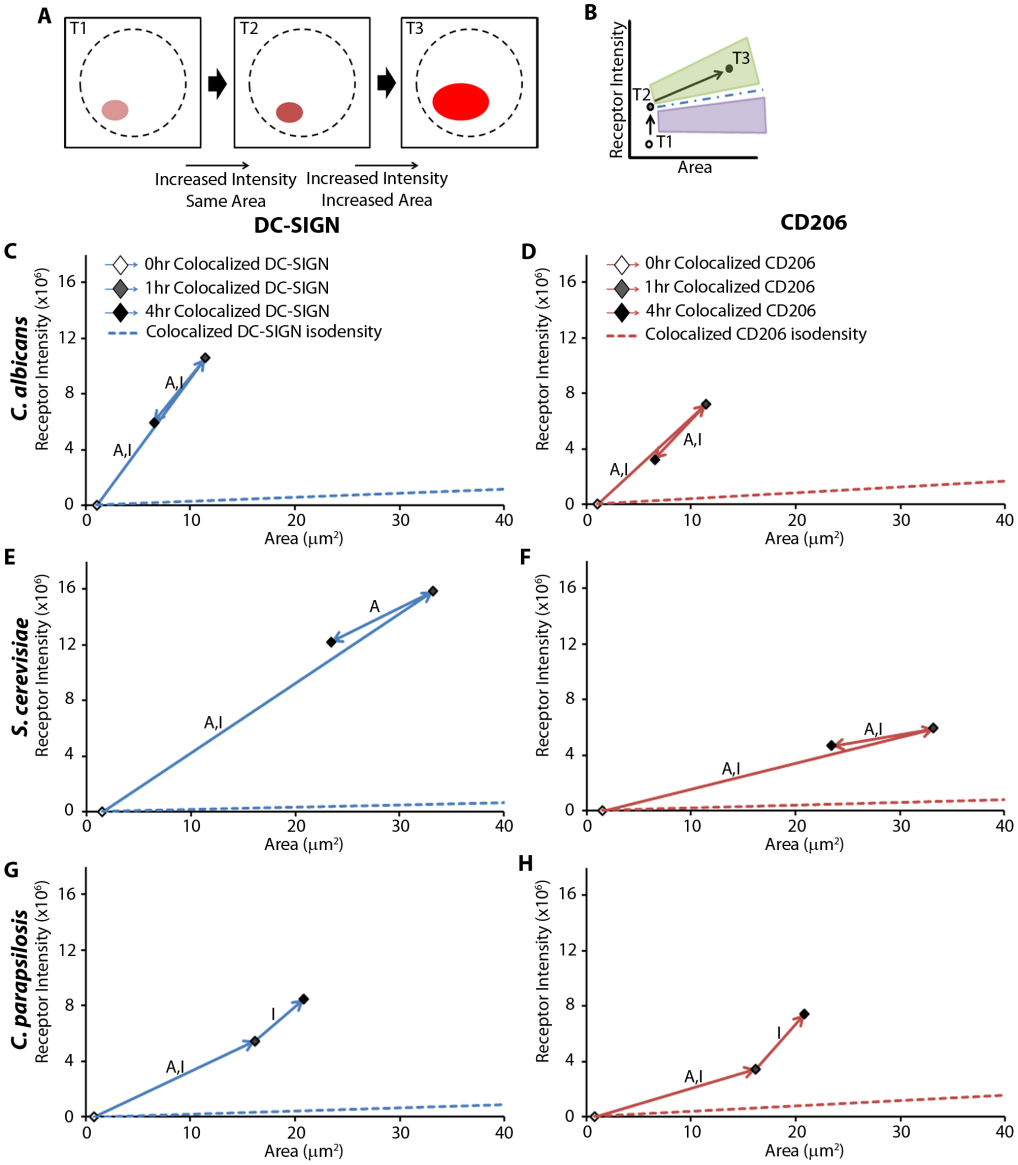
Receptor density. A) A schematic illustration of how temporal changes in receptor region area (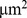) and intensity are reflected. B) During the 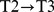 time period, both receptor intensity and area increase. The blue dashed line illustrates the isodensity line at T2. 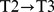 slopes greater than this isodensity line (i.e., illustrated by the green shaded area) represent intensity and area changes resulting in increasing density over this time period. Conversely, 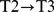 slopes less than the isodensity line (i.e., illustrated by the purple shaded area) would result from decreasing density over this time period. C–H) Distribution patterns of change in contact site area and intensity for DC-SIGN (C,E,G) and CD206 (D,F,H). Diamonds denote colocalized regions. Arrows annotate the temporal connection of the datapoints: blue for colocalized receptor regions. Time step arrows labeled “A” exhibit a significant change in area at 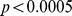. Time step arrows labeled “I” exhibit a significant change in intensity at 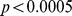. Dashed lines (nearly on top of the Area axes in (C–H) indicate the isodensity lines for zero hour colocalized datapoints. Statistical significance was determined by ANOVA and Tukey's post-hoc test with 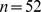 samples per donor for 1 and 4 hours and 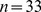 samples per donor for 0 h.

The clustering of receptors at cell-cell contacts is a common theme in immunoreceptor signaling, and this mechanism drives the formation of membrane regions with increased receptor density. Receptor density is one factor that can regulate the efficiency of signal transduction and membrane trafficking of the receptor. Because receptor density in the contact is coordinately defined by the total amount of receptor recruited and the membrane area that it occupies, we created density graphs to display the difference between colocalized and single-positive DC-SIGN and CD206 distributions (Fig. 5).

Fig. 5A provides a schematic example of contact site density over three time points (T1-3), and Fig. 5B provides the corresponding density graph analysis. At T1, there is a small area with a small amount of intensity within that area that increases in intensity but not area in T2 (thus, higher density in T2 vs. T1). At T3, this region exhibits increases in area and intensity. The dashed “isodensity” line depicts the set of all combinations of intensity and area with the same density as at T2. Thus, because the 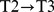 slope is greater than that of the isodensity line (i.e., T3 lies in the green shaded area), the 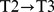 transition involves an increase in density at T3 relative to T2. This would not be immediately apparent without reference to the isodensity line.

In colocalized regions (where both DC-SIGN and CD206 are found within the same voxel), we found that *C. albicans* accumulated the highest density for both DC-SIGN and CD206 within the first hour (Fig. 5C,D). The same trend was also found in *S. cerevisiae* and *C. parapsilosis*, but with somewhat lower CTL densities achieved (Fig. 5E,F,G,H).

The development of a pronounced colocalized region with high receptor density could promote receptor cross-talk and strong adhesion. When we compared fungal species to one another, we found that *C. albicans* accumulated 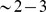 times more colocalized DC-SIGN density than *S. cerevisiae* and *C. parapsilosis* at the first hour (Fig. 5C,E,G), but interestingly *C. albicans* accumulated 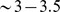 times more colocalized CD206 than *S. cerevisiae* and *C. parapsilosis* (Fig. 5D,F,H). Contact sites with *S. cerevisiae* and *C. albicans* both reduced their CTL colocalized density between the first hour and fourth hour (Fig. 5C,D,E,F), whereas *C. parapsilosis* likewise gained density but did not exhibit an area or intensity loss at longer duration (Fig. 5G,H). We note that all contacts increased their receptor density greatly in the first hour (slopes well above the stated isodensity line), but *C. albicans* contacts were notable for being dense because they recruited DC-SIGN and CD206 well but remained small in area.

Prior to our detailed analysis of the contact sites, we used the Manders coefficients to estimate the degree of colocalization. The coefficient M1 (the proportion of DC-SIGN colocalized with CD206) indicated very high degrees of colocalization in 1 and 4 hour contacts for all three yeast species and both CTLs. As the Manders coefficients are influenced by both degree of overlap and intensity, they are not completely specific for variations in the amount of colocalization. Our contact site analysis provides more detailed results on colocalization in general. In this case, the Manders analysis and our contact site analysis of colocalization agreed with one another in finding predominant colocalization in contacts under all tested conditions.

### Binding and Phagocytosis Efficiency

**Figure 6:**
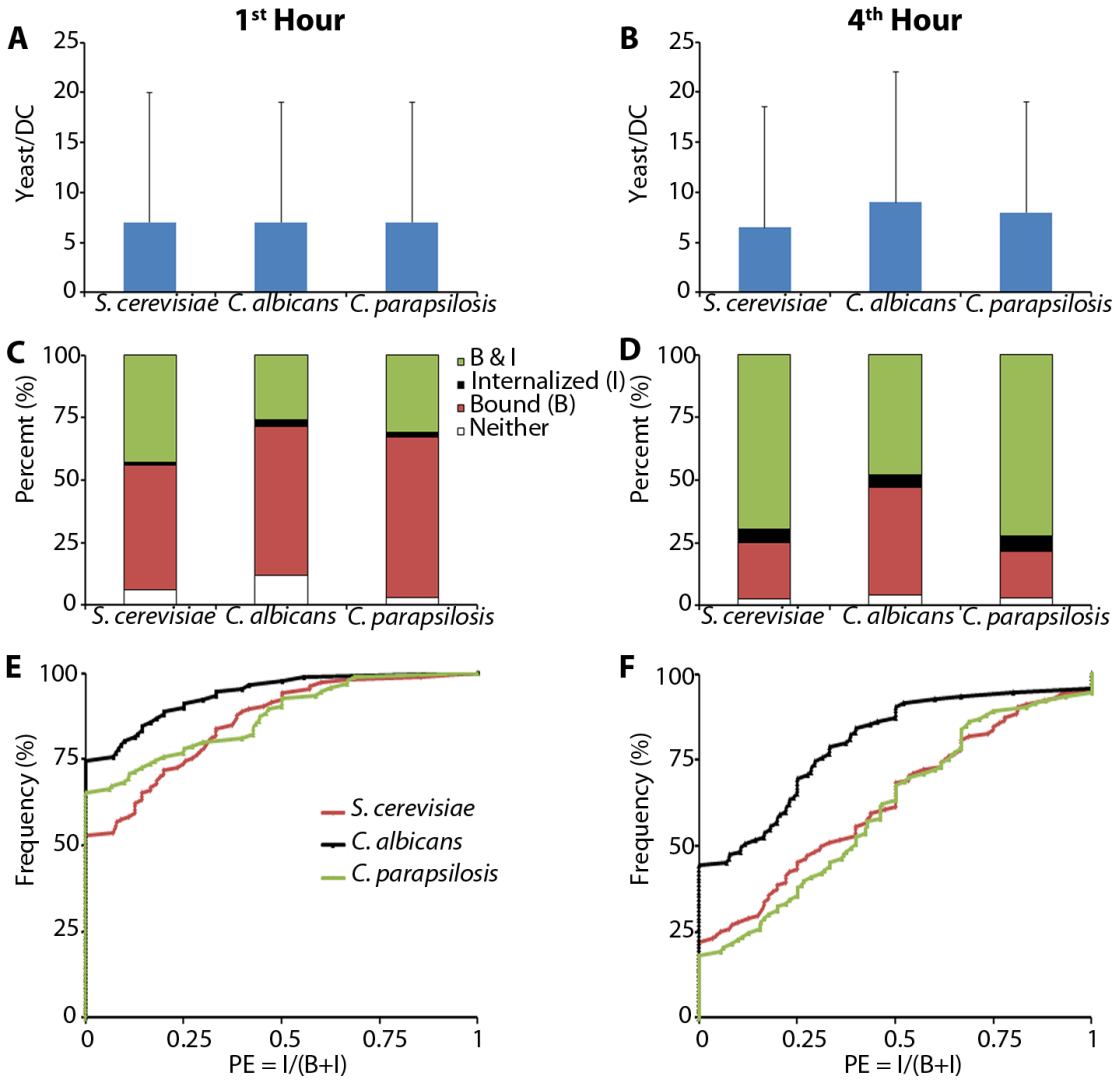
Efficiency of binding and engulfment. A,B) The median number of yeast binding per DC at 1 (A) or 4 (B) hours is not significantly different between the three species tested. C,D) Distribution of categories at 1 hour (C) and 4 hours (D) for all yeasts. E,F) Cumulative probability distributions of phagocytic efficiency (PE) at 1 (E) and 4 (F) hours show that DC populations binding *C. albicans* exhibit more DCs failing to engulf any yeast and skewing to lower PE values than for those that do internalize yeast. Statistical comparison of PE at 1 hour displayed significant differences (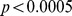) between *C. albicans* and *S. cerevisiae*, and at 4 hours between *C. albicans* and both of the other yeasts. Statistical significance was determined by the Mann-Whitney test. Data in A and B are presented as the median and interquartile range pooled over 3 independent repeats with different donors, and within each repeat, 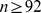 DCs sampled for each experimental condition.

We hypothesized that the differential spatiotemporal patterns of receptor recruitment that we observed for *S. cerevisiae*, *C. albicans*, and *C. parapsilosis* would be correlated with the functional differences in binding and/or phagocytic efficiency during DC-yeast interaction. In particular, the smaller area contacts observed for *C. albicans* were suggestive of less actin reorganization and pseudopod extension. We quantified binding and phagocytic efficiency for DCs treated with yeasts for 1 and 4 hours, as described in the methods section. Interestingly, there was no significant difference in the median number of yeasts captured per DC between species at 1 or 4 hours (Fig. 6A,B). We categorized DCs based on their interaction with yeasts as “neither” (no bound or internalized yeast; excluded from analysis), “bound” (only surface bound yeast), “internalized” (only internalized yeast), and “B&I” (some bound and some internalized yeasts). Despite this equivalent capture of yeasts, we found that DC populations exposed to *C. albicans* were skewed to distributions that reflected lower levels of internalization (i.e., decreased percent of the population in the “B&I” category) relative to that seen for DCs exposed to *S. cerevisiae* or *C. parapsilosis* (Fig. 6C,D). To understand this phenomenon in more detail, we examined cumulative probability distributions of phagocytic efficiency (PE) for DCs exposed to all three yeasts over 1 or 4 hours. We found that the proportion of DCs that failed to internalize any bound yeast (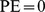) was higher for *C. albicans* than the other species for both time points (Fig. 6E,F). Furthermore, of those DCs that did internalize some yeasts (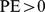), these DCs exhibited generally lower phagocytic efficiencies for *C. albicans* than other species. These trends represented a significant difference in PE distributions for *C. albicans* versus *S. cerevisiae* at 1 and 4 hours, and a significant difference between *C. albicans* and *C. parapsilosis* at 4 hours. The distribution of PE values was not significantly different between *S. cerevisiae* and *C. parapsilosis* at either time.

## Discussion

The analysis tool that we developed allows quantification of receptor behavior on an approximately spherical surface extended across multiple 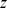-axis confocal sectioning depths. This capability, coupled with the ability to resolve and quantify receptor structures on this host-pathogen contact site surface, allowed us to discern interspecies differences in CTL mobilization and organization during fungal recognition by dendritic cells. Despite the presence of abundant 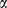-mannoside ligands of DC-SIGN and CD206 in the cell walls of all fungi tested, we observed dissimilar spatiotemporal patterns of receptor recruitment amongst *S. cerevisiae*, *C. albicans* and *C. parapsilosis*. DCs recruited DC-SIGN and CD206 to contact sites with all three yeast species to achieve tens to over a hundred fold enrichment of receptors. However, receptor recruitment peaked earlier for *C. albicans* and *S. cerevisiae*, while *C. parapsilosis* contacts developed in a slower, progressive manner. Also interesting was the observation that *S. cerevisiae* contacts were quite large while *C. albicans* contacts were notable for being the smallest at both one and four hours. Because contact site area is likely to reflect the success of cytoskeletal remodeling in response to fungal recognition, we examined whether receptor recruitment patterns or contact site area characteristics correlated with the functional outcome of phagocytosis. We found that, despite similar ability to capture all yeasts, DCs exhibited significantly lower phagocytic efficiency when challenged with *C. albicans* in comparison with *S. cerevisiae* and *C. parapsilosis*. These data suggest that strong contact site recruitment of mannan-binding CTLs is important for capture of fungi by DCs, which is consistent with the fact that mannan is the dominant ligand on the cell wall surface. However, intensity of DC-SIGN or CD206 recruitment is not a strong predictor of phagocytic outcome. For instance, *S. cerevisiae* recruited the most DC-SIGN at one hour, while the intensity of DC-SIGN in *C. parapsilosis* contacts was much slower to develop to similar levels, but both yeasts were well-phagocytosed with similar efficiencies. Contact site area was a good predictor of phagocytic efficiency, and it is likely that both readouts reveal a relative paucity of cytoskeletal response to *C. albicans* yeast relative to *S. cerevisiae* or *C. parapsilosis*. This could reflect the existence of cell wall features possessed by *C. albicans* that minimize phagocytosis and aid in partial evasion of the innate immune response.

These differences in spatiotemporal distribution patterns may result from subtle differences in the fine structure of mannan. *C. albicans* mannans have been shown to contain structural features such as *β*-(1,2)-linkages and branching *α*-linked oligomannoside side chains [55], [56] which are not shared by *S. cerevisiae* or *C. parapsilosis*. Mannan structural differences can influence the antigenicity and surface chemistry of the cell wall [57], [58].

In contrast to other cell-cell contact signaling systems with more laterally mobile ligand/receptor pairs (i.e., the immunological synapse), the ligands presented by the fungal cell wall are part of a dense and highly interconnected network. Although the cell wall does undergo remodeling, the lateral mobility of polysaccharide ligands in the contact site is quite low. Interestingly, recent work from Dufrêne and Lipke and colleagues has demonstrated that important mannoproteins of the Als adhesin family can be reorganized into distinct 100–500 nm amyloid domains in the cell wall of *C. albicans* upon application of force, and changes in Als protein exposure and organization are also seen under conditions such as hyphal germination and treatment with echinocandin drugs [59]–[62]. The consequent spatial reorganization of mannan ligands could be important for the nanoscale organization of DC-SIGN and CD206 in contact sites with DCs. Als adhesins are anchored to fibrillar glucan in the cell wall and above referenced results suggest that their mobility in the cell wall consists of gyration about their anchorage points, not long-range lateral mobility. However, some mannoproteins are known to be non-covalently associated with the cell wall and these could possess greater lateral mobility. In our analysis of fungal contact sites, we saw that receptors congregated in specific, micron-scale membrane structures despite presumed low levels of ligand lateral mobility. This study utilized fixed yeasts to provide more controlled experimental conditions and more straightforward data interpretation. This simplification precludes mannoprotein mobility during DC-yeast interaction, so future experiments in live cell interaction systems will be necessary to fully elucidate the role of fungal cell wall reorganization in these host-microbe interactions.

The organization of receptors into micron-scale membrane substructures, wherein transmembrane protein populations may mix and achieve altered density, will likely influence the efficiency and maintenance of signal transduction. A previous report describing the “phagocytic synapse” showed that the lateral reorganization of the CTL Dectin-1 and the phosphatase CD45 influences Dectin-1 signaling [63]. The mechanisms that drive the formation of specific membrane structures in fungal contacts, such as ligand patterning on cell wall surfaces, observed for patches of 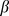-glucan exposure on *C. albicans* [24], [64], are an interesting topic for future research.

CTLs have been described to exist in DC membranes as discrete nanodomains of approximately 80–100 nm diameter by several imaging methods such as transmission electron microscopy, near-field scanning optical microscopy and super resolution fluorescence imaging [45], [65]–[68]. These domains have interesting biophysical properties, such as a lack of exchange of receptor with the surrounding membrane and nearly complete segregation of DC-SIGN and CD206 nanodomains in resting DC membranes [68]–[70]. Recently, we have observed that nanoscale organization of CTLs in fungal contacts is altered relative to non-contact membrane in favor of less individual nanodomain structure and more longer-range nanostructure, consistent with close packing of domains (unpublished data, AKN). The significance of receptor colocalization and changes in receptor density in contact sites is that spatial proximity influences signal transduction by increasing amplitude and persistence of signaling as well as promoting crosstalk between receptors. Application of our analysis tool to higher resolution imaging modalities, such as Stimulated Emission Depletion microscopy and 3D direct Stochastic Optical Reconstruction Microscopy, may provide insights into critical early receptor rearrangement events in innate immune fungal recognition in future studies.

Cell-cell contacts are a common theme in biology, being integral to such diverse processes as lymphocyte activation, tissue development and neural communication. Therefore, we anticipate that this tool will have broad utility in other fields where quantification of receptor and/or organelle mobility relative to a cell-cell contact is needed. Some examples of other potential biomedical applications include other phagocytic synapses (i.e., macrophage scavenging of apoptotic bodies), the immunological synapse between T cell and antigen presenting cell, receptors within the synapse between neurons, the association between plasma membrane and SNARE complexes on the ER for calcium signaling, between 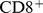 cytotoxic T cells or NK cells and virally infected target cells, and B cell or mast cell activation by particulate antigen. Much information can be derived from standard confocal optical imaging, as we have demonstrated. However, promising progress in techniques for 3D super resolution microscopy should provide access to structural detail on at least a log-order higher resolution, and such data could be analyzed by our method to assess changes in biologically significant structures such as receptor microclusters and STIM/Orai mediated 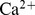 signaling microdomains.

## Materials and Methods

### Fungal Culture

*C. albicans* (ATCC, Manassas, VA, #MYA-2876), *C. parapsilosis* (ATCC, Manassas, VA, #22019), and *S. cerevisiae* (ATCC, Manassas, VA, #26108) were cultured in YPD broth in an orbital incubator at 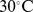 until exponential phase growth. Prior to application to dendritic cells, yeasts were fixed with 2.5% PFA at room temperature for 20 min followed by extensive PBS washing.

### Tissue Culture

We obtained human peripheral blood leukocytes from discarded leukocyte reduction filters provided by United Blood Services of Albuquerque. The filters were back-flushed with 300 mL HBSS, and the collected cells were spun over Ficoll-Paque Plus (GE Healthcare, Sweden, #17-1440-02). Monocytes were purified by adherence on tissue culture flasks. Immature dendritic cells were prepared by differentiation of monocytes in RPMI supplemented with 10% FBS, 1% Penicillin/Streptomycin, 10 mM Hepes, and 1 mM sodium pyruvate, 500 IU/mL human IL-4 (Peprotech, Rocky Hill, NJ, #200-04) and 800 IU/mL human GM-CSF (Sanofi, Bridgewater, NJ, Leukine/sargramostim/) at 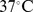, 5% 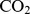 for 7 days. Immature DCs existing in 7 day cultures were exposed to the specified yeasts (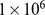 per sample) for the specified times. These conditions were found to represent a relatively light challenge for DCs with yeast that is unlikely to overwhelm the ability of DCs to bind yeast, recruit receptors to contact sites or engulf particles. This use of human blood products was reviewed and approved by the University of New Mexico Health Sciences Center Human Research Review Committee.

### Immunofluorescence Analysis

Fixed specimens were blocked and stained with primary and secondary antibodies. Primary antibodies were as follows: anti-human CD209 (BD Pharingen, San Diego, CA, #551186) and anti-MRC1 (Abnova, Taiwan, #H00004360-M02) applied at a concentration of 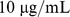 for 30 minutes at 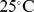. These conditions provided an excess of primary and secondary antibodies and achieved saturation binding of receptors. Identical staining conditions were used in the preparation of all samples for contact site analysis. The following secondary antibodies were used: Alexa Fluor 488 goat anti-mouse (Invitrogen, Grand Island, NY, #A21141) and Alexa Fluor 647 goat anti-mouse (Invitrogen, Grand Island, NY, #A21240) applied at a concentration of 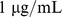 for 30 minutes at 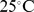. Cell membrane was visualized by Cell Mask Orange (CMO) (Invitrogen, Grand Island, NY, #C10045) at a concentration of 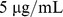 for 5 minutes at 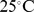. This staining condition allows only DC membranes to stain. The CMO staining duration is insufficient to allow dye penetration of the cell wall for yeast plasma membrane staining. Fully phagocytized yeasts were not accessible to receptor staining and are thus not represented in contact site receptor analysis. Contact sites randomly chosen for analysis of receptor spatiotemporal distributions exhibited a range of expected engulfment morphologies.

Fluorescent proteins and lipids were imaged with a FV1000 laser scanning confocal microscope (Olympus, Center Valley, PA) equipped with a 60×, 1.42 NA, Plan-Apochromat oil immersion objective. AlexaFluor488 (reporting the distribution of CD209) was excited with a 15 mW, 473 nm diode laser operated at 1% power; AlexaFluor647 (reporting the distribution of CD206) was excited with a 20 mW, 635 nm diode laser operated at 1% power, and CMO (reporting the dendritic cell membrane 3D profile) was excited with a 15 mW, 559 nm diode laser operated at 1% power. These lines were reflected to the specimen by a 405/473/559/635 multi-edge main dichroic element, and emission was routed through the main dichroic mirror and confocal pinhole (115 nm diameter) to secondary longpass dichroics (or a mirror) followed by bandpass emission filters in front of 3 independent PMT detectors. Specifically, the emission light passed by the main dichroic was directed to PMT1 (AF488/DC-SIGN channel) via reflection from a longpass 560 nm cutoff dichroic mirror and passage through a BA490-540 nm bandpass filter. Emission passing this dichroic was directed to PMT2 (CMO channel) via reflection from a longpass 640 nm cutoff dichroic mirror and passage through a BA575-620 nm bandpass filter. Finally, emission light passed through this dichroic was directed to PMT3 (AF647/CD206 channel) via reflection from a mirror and passage through a BA655-755 nm bandpass filter. Z-stacks were recorded with 250 nm spacing. Other parameters were pixel dimensions (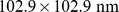 square pixels in the 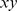 dimension), pixel dwell time (2 µs/pixel), detector sensitivity (PMT1 640 volts; PMT2 455 volts; PMT3 610 volts; gain = 1 and offset = 0 for all PMTs). All imaging parameters as described above were kept constant during acquisition of all images for contact site analysis. Photo bleaching was not found after examining the 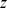-axis profile of 0 hr time points, 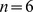. Each experimental result presented in this work represents pooled data from independent replicates with DCs from three separate donors. Within any given replicate, DCs were chosen at random for imaging and analysis. We imaged a minimum of 50 contact sites per species per time point for contact analysis. Statistical significance was determined by ANOVA, Tukey post-hoc test.

### Phagocytosis Efficiency

Fixed yeast were stained with two different markers prior to being added to the live DC culture. The first marker was Calcofluor White (Sigma-Aldrich, St. Louis, MO, #F3543) at a concentration of 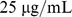 for 20 minutes at 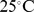. The second label was Biotin-NHS (Sigma-Aldrich, St. Louis, MO, #H1759), which was conjugated to cell wall proteins of yeasts (fixed with paraformaldehyde, as above) at a concentration of 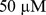 for one hour at 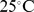 in PBS at 8.5 pH. After staining, these yeast particles were added to live DC culture for either 45 minutes or three hours and 45 minutes. At either time point, 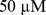 streptavidin-Alexa Fluor 647 (AF647) in RPMI warmed to 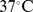 was added to the live DC culture for 15 minutes. At this point the DCs were fixed with 4% PFA in PBS for 10 minutes at 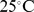 followed by extensive PBS washing.

Fixed yeast particles were imaged with a FV1000 laser scanning confocal microscope (Olympus, Center Valley, PA) equipped with a 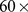, 1.42 NA, Plan-Apochromat oil immersion objective. Calcofluor White (marker for all yeast) was excited with a 50 mW, 405 nm diode laser operated at 1% power and streptavidin conjugated Alexa Fluor 647 (marker for only external yeast) was excited with a 20 mW, 635 nm diode laser operated at 1% power. These lines were reflected to the specimen by a 405/473/559/635 multi-edge main dichroic element, and emission was routed through the main dichroic mirror and confocal pinhole (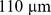 diameter) to secondary longpass dichroics (or a mirror) followed by bandpass emission filters in front of 2 independent PMT detectors. Specifically, the emission light passed by the main dichroic was directed to PMT1 (fluoresce brightener channel) via reflection from the mirror and passage through a BA430-455 nm bandpass filter. Emission passing this dichroic was directed to PMT3 (streptavidin conjugated to AF647 channel) via reflection from a mirror and passage through a BA655-755 band pass filter. Z-stacks were recorded with 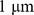 spacing. Other parameters were voxel dimensions (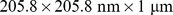 voxels in 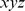 dimensions), pixel dwell time (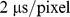), detector sensitivity (PMT1 650 volts, PMT3 570 volts); gain = 1 and offset = 0 for all PMTs). All imaging parameters as described above were kept constant during acquisition of all images for phagocytosis assay.

Bound and internalized yeast were enumerated manually on a per DC basis in all 3D confocal datasets. Bound yeasts were identified based on their location on DCs (DIC) and positive signal for both Calcofluor White and AF647 emission. Internalized yeasts were identified by apparent localization inside a DC (DIC) emission in the Calcofluor White channel only. We calculated the median and interquartile range for both categories over all DCs imaged. Phagocytosis Efficiency (PE) for each DC was calculated as the number of yeasts that were identified as internalized divided by the total number of yeasts associated with the same DC (that is, surface bound plus internalized yeasts). Statistical significance was determined by the Mann-Whitney test.

### Quantitative Analysis of Contact Sites

To facilitate the quantitative analysis of the contact sites, we developed a graphical user interface for the analysis programs. This interface allows the user to load the image files, specify parameters and select regions of interest, for example, see the first row in Fig. 2. The images in this row show a few dendritic cells interacting with yeast cells. The user selects a yeast cell for analysis by clicking on it, which spawns a new window with a close-up view of the selected region. In the image of a single yeast cell, the user selects the center of the yeast and an inner and outer radius such that the yeast cell wall surface lies between the spheres determined by the two radii. The contact site is assumed to reside within this spherical shell and surfaces that depart significantly from sphericity (e.g., nearly planar regions) can still be analyzed as long as the contact site falls within the spherical volume described by the two radii. The underlying analysis programs then transform the data to spherical coordinates and project the intensity values onto the outer spherical surface which approximates the yeast cell surface. The area of membrane/cell wall contact between the dendritic cell and yeast cell is identified by thresholding, and receptor fluorescence intensities and analysis results are written to a spreadsheet for further analysis. We have validated our method against artificial objects where the recovered receptor intensities, locations and colocalizations can be compared with our knowledge of the ground truth for these parameters.

In more detail, our data sets have four channels:

channel 1, DC-SIGN (green, G),
channel 2, membrane stain (CMO),
channel 3, CD206 (red, R),
channel 4, transmitted light (DIC).

The red and green channels are intensities from two different fluorophores. For each channel, the data are the intensities of the light emitted in each voxel of a three dimensional image (Z-stack). The transmitted light channel images of a few dendritic cells and several yeast cells are shown in Fig. 2. These images are used to select a single yeast in contact with a dendritic cell that is to be analyzed. Using the interface, the user selects the center of the yeast and then draws two radii, 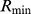 and 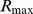, that determine two spheres such that the voxels between the two spheres contain all of the light emitted from the contact site.

**Figure 7:**
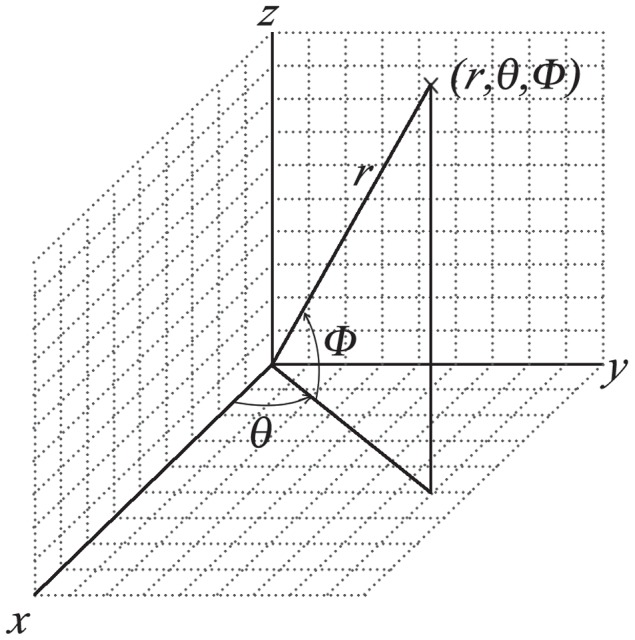
Geographical spherical coordinates.

**Figure 8:**
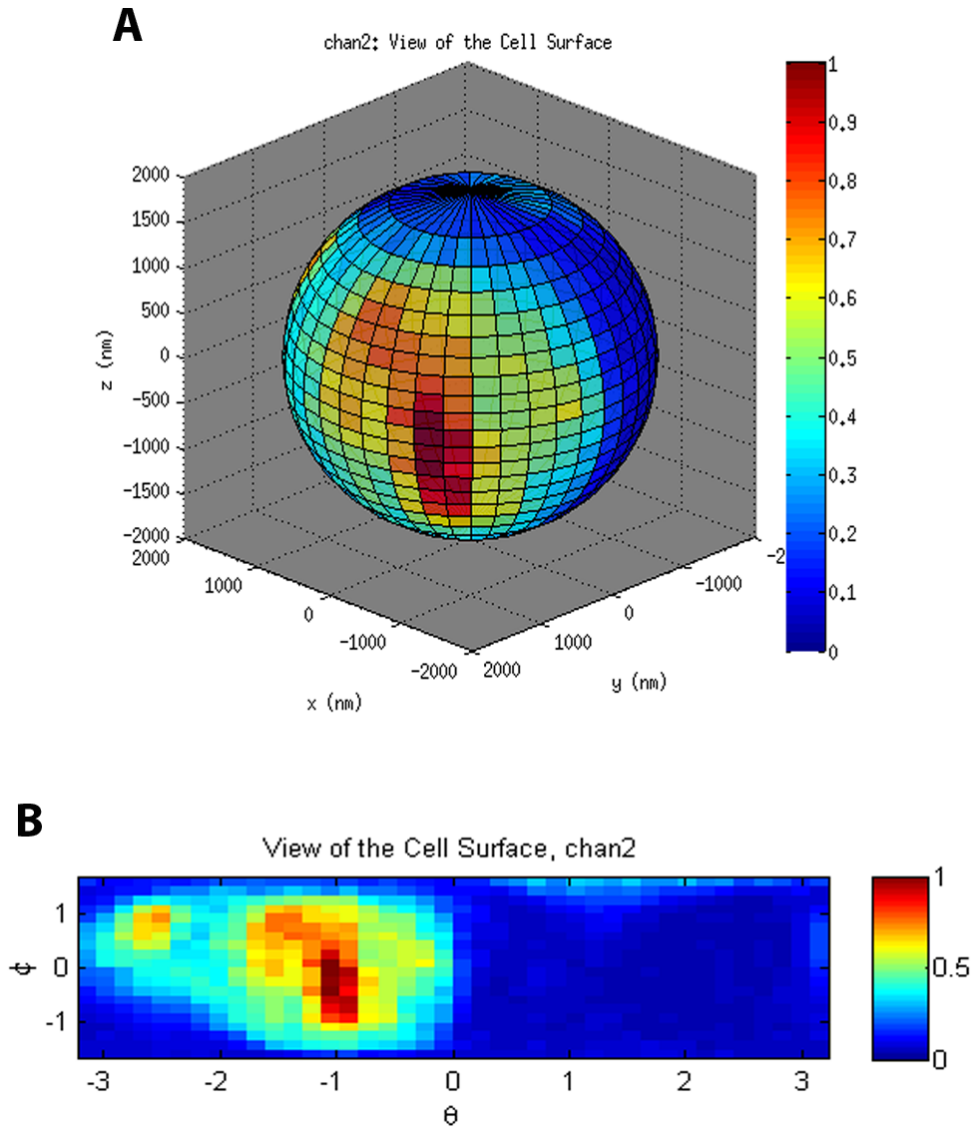
Spherical representation (A) and planar representation (B) of the normalized surface intensity of the membrane stain (CMO) channel of the contact site. Note that the pixels are of uniform area.

To analyze these data, the program establishes spherical coordinates (see Fig. 7) with origin at the center of the yeast cell. These coordinates are used to divide the space up into spherical voxels, and additionally divide the surface of the larger of the two spheres (with radius 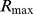) into pixels as shown in Fig. 8A. We analyze the data for each color by first transferring the intensities from the rectangular to the spherical voxels. This is done by dividing all of the relevant rectangular voxels into many much smaller rectangular subvoxels, and then apportioning the intensities among the subvoxels. For each subvoxel, the spherical voxel that contains the center of the subvoxel is determined, permitting the intensities from that subvoxel to be transferred to the appropriate spherical voxel. This transfer is computationally expensive, so several techniques were developed to make this process more efficient. See below for further details.

The localization/colocalization analysis starts with a spherical projection of the intensities viewed as periodic data in a rectangle; see Fig. 8B. The analysis program uses thresholding to identify the contact site and performs several standard image processing techniques to prepare the images for further processing. The analysis program selects a region containing the contact site and then determines the spherical pixels occupied by receptors, presents the results to the user, and also writes a spreadsheet file that can be used for additional analyses.

#### Spherical approximation

As noted, the user interface provides us with a three dimensional rectangular volume is made of 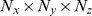 rectangular voxels that have dimensions 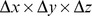. All lengths are in nanometers (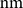). The interface also provides us with an estimate 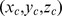 of the center of the yeast cell. We introduce spherical coordinates (Fig. 7) centered at 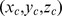:
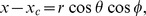


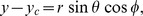


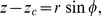
where 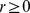, 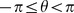 and 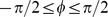. The interface also provides two user-supplied radii, 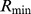 and 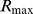, such that the light emitted by fluorophores on the dendritic cell membrane satisfies 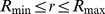. The two spheres are given by
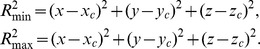



**Figure 9:**
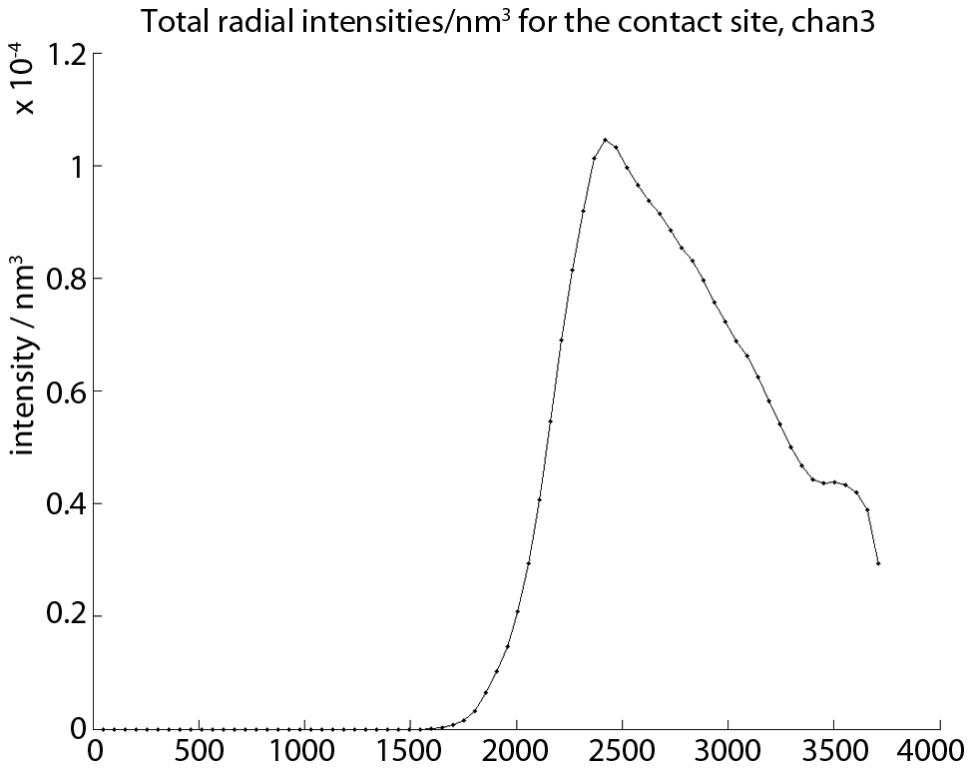
The total intensity per volume as a function of 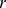 for a user specified spherical shell. The rapid rise and descent is characteristic for a contact site contained wholly within the designated volume.

To ensure that the contact site really resides with the specified spherical shell, the averaged and total intensities per volume for each channel are plotted as a function of 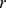 (the values are collected over thin concentric spherical shells). The density of the total intensity (and to a lesser extent, of the average intensity) shows a characteristic spike between 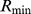 and 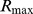 when the contact site is contained within the user selected region. An example of these plots is illustrated by Fig. 9. In addition, we color code the radii of maximum intensity per spherical surface pixel over the range 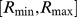, and then plot the results for each channel. These pictures resemble the bottom plot in Fig. 8, but with somewhat different scaling.

We next create spherical voxels based on the center and radius of the larger sphere. Let 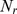, 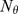 and 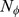 be the number of voxels in the 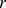, 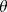 and 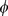 directions. We choose uniformly sized voxels in the 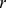 and 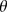 directions. If we choose the size of the voxels in the 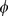 direction to be evenly spaced, then the pixels on the spherical surface 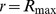 (or any other radius) will have a much smaller area at the poles (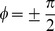) than at the equator (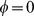). A better choice is to set the spacing in 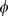 to so that the pixels on the spherical surface all have the same area. In addition, we will need the volume of a spherical voxel later.

Consider a spherical voxel described by 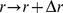, 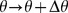 and 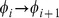. Its volume is
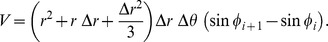
The area of a surface pixel at 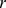 is then given by
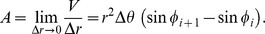
Let 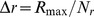 and 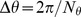. If we choose
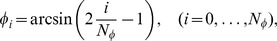
then the area of the spherical surface pixels at 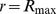 will be
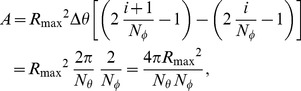
which depends only on the radius 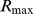 and the numbers 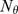 and 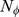. Thus, all of the areas will be equal as illustrated in Fig. 8.

The approximate size of the rectangular voxels are 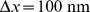, 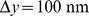 and 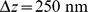, so that the volume of such a voxel is 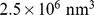. The radius of the yeast cell is approximately 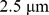, so if we choose 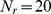, 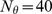 and 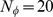, then the spherical voxels near the surface of a sphere with that radius have a volume of about 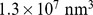, while the area of a pixel on the sphere's surface is 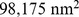.

The basic algorithm that transfers the intensities from the rectangular voxels to the spherical voxels proceeds by first dividing the rectangular voxels into 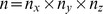 subvoxels. To make the subvoxels cubes, we use the aspect ratio 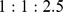 for 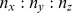. The intensity 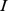 of the light emitted by the fluorophores in a rectangular voxel is equidistributed as 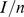 into the subvoxels. The light intensity coming from each subvoxel is then added to the intensities in appropriate spherical voxels containing the centers of these subvoxels.

A quick way to find the spherical voxel that contains a subvoxel's center is to first transform the center to spherical coordinates using 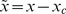, 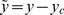, 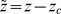, and then set
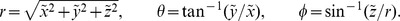
Recall that 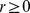, 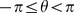 and 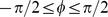. First, set 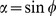, so that 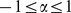, 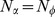 and 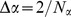. Then the indices of the spherical voxel are given by
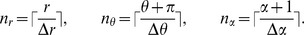
The ceiling function 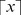, 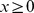, gives the smallest integer larger than or equal to 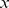.

By far the most computationally expensive part of the analysis is moving the intensities from rectangular to spherical coordinates. The number of spherical voxels does not have much impact on the running time of the code. However, note that if we double the values of 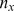, 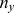 and 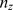, then 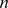 is multiplied by 8, so the transfer will cost a factor of 8 more in computation time. To reduce this cost, we only consider rectangular voxels that are close to the yeast cell surface.

We note that the greatest error in the rectangular to spherical conversion occurs for voxels when 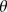 or 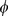 is near 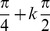 for 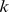 an integer, where the spherical surface slices diagonally through the rectangular voxels. Instead of doubling the number of subvoxels throughout, we can reduce this error by increasing the number of subvoxels per rectangular voxel near these angles. In our conversion routine, we change the refinement in the appropriate direction as a function of 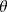 or 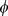 by first detecting the distance the current angles are from 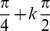:
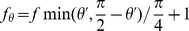


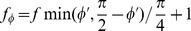
where
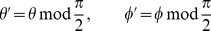
and 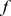 is the increase factor, such that 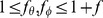. After some experimenting, we choose 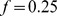. Then, the number of subvoxels in each direction for our examples become
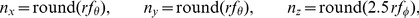
(1)where 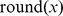 gives the nearest integer to 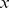, and 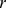 (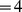 in our examples) is the minimum refinement factor. The ratio 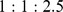 for 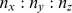 is chosen to correspond to the rectangular voxel dimensions as noted above.

The maximum intensity in the radial direction between 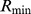 and 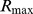 is taken to be the cell membrane. We exclude intensities about a 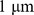 range from this surface to ensure analysis is performed only for activity at the cell membrane. The intensities from the spherical voxels are transferred to spherical surface pixels using weighted sums of the voxels along the same radial direction. The weights are the volumes of the voxels. The surface intensities can be plotted on the sphere or in a rectangle in the plane as in Fig. 8. These figures were created using 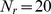, 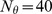, 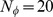 and 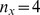, 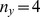, 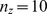 (but as modified by Equation 1) to subdivide the rectangular voxels and to transfer the intensities to spherical voxels. This projection makes it practical to accurately estimate the amounts of localization and colocalization of the receptors.

Detailed formulas are available in the implemented computer codes. All algorithms and code were validated using constructed model problems. All analysis codes are written in MATLAB and image reading and display functionality is provided by the Bioformats [71] and DIPimage [72] toolboxes. All programs are available from: http://stmc.health.unm.edu/

#### Contact site analysis

The interface passes the data structure containing the surface intensities in spherical coordinates to the contact site analysis function. The purpose of this function is to perform multiple spatial analyses which will allow biologists to draw conclusions about the behavior of dendritic cell membrane proteins in contact with yeast cells. The analysis is performed in the following steps:

1. Automatic thresholding to identify the contact site.
2. Background subtraction.
3. Find the voxels containing a signal from the red channel, the green channel, or both channels.
4. Calculate the proportion of the total contact site area occupied by each signal population.
5. Quantify intensity of each signal population.
6. Calculate Manders' Coefficients.
7. Output all workspace variables to a file.

**Figure 10:**
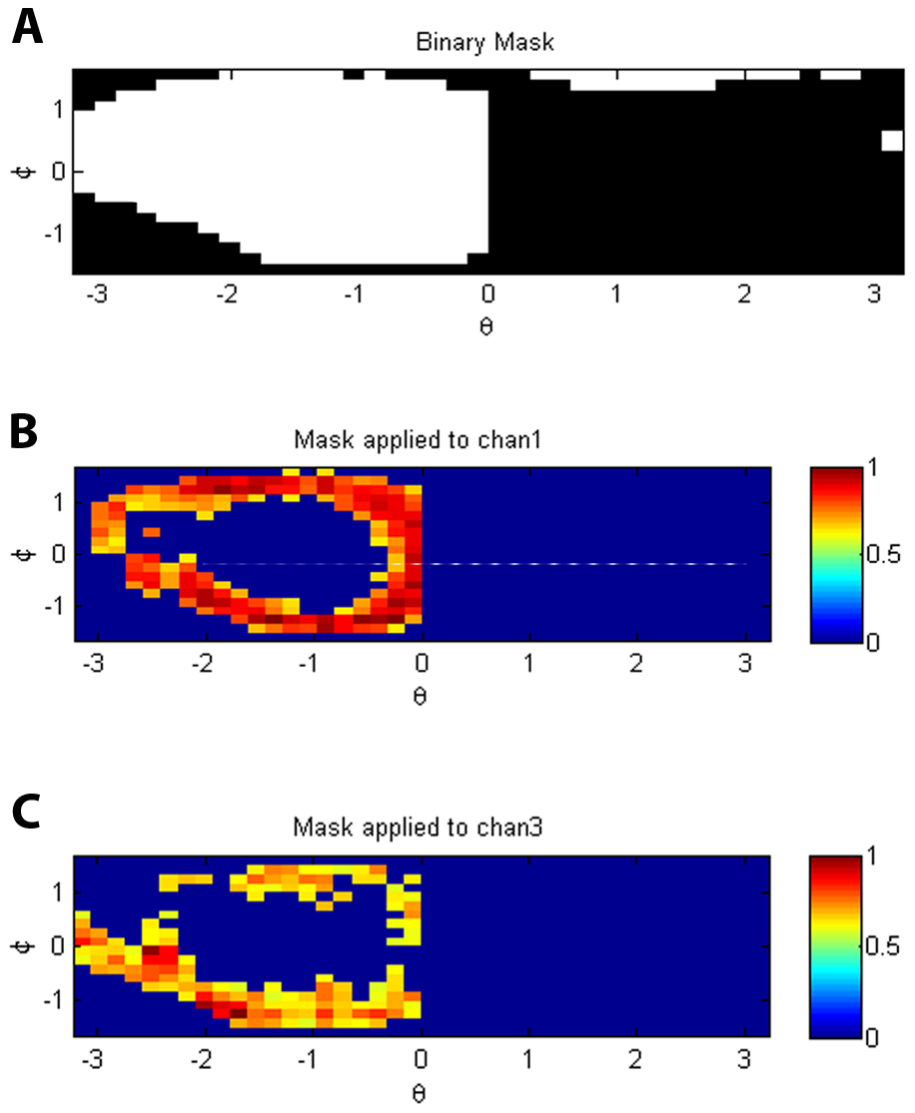
Masking. (A) Binary mask created by thresholding the membrane stain surface intensity data shown in Fig. 8. (B,C) The two fluorescence channels to be compared (henceforth referred to as Red and Green) after mask application and background removal.

Thresholding is performed on the membrane stain channel (Fig. 8), using a threshold that is based on the mean intensity. The purpose of the thresholding step is to isolate the area consisting of 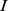 voxels in which there is strong membrane stain signal, indicating a site of contact between the yeast cell and the dendritic cell. We note that the receptor channels are also thresholded in the same manner as the membrane stain channel, but in the original rectangular voxel space (so before conversion to spherical voxels), which we found to be optimum for our data. Thresholding results in a binarized image (Fig. 10A), which is used as a mask defining the yeast-dendritic cell contact site. The newly created binary mask is then applied to the remaining two fluorescence channels (Fig. 10B,C).

Next, the red and green fluorescence channel intensity values, 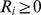 and 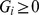 for each voxel 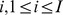 in the defined contact site, are used to assign voxels to one of the following groups: voxels in which signal from both fluorescence channels are present, voxels containing only signal from one fluorescence channel or the other, or voxels containing no signal. For 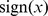 given by 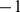 if 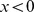, 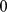 if 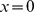 and 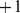 if 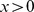, the number of voxels in each group are defined by
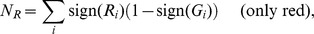


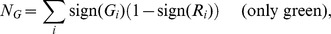


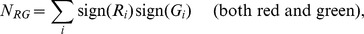


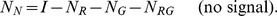



The proportions of the total contact site occupied by each group is then 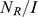, 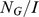, 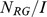 and 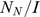. The intensities in the fluorescence channels are given by
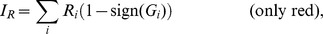


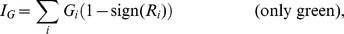


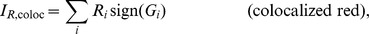


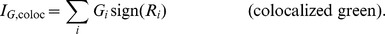
With this information available, it is easy to compute other values that measure localization and colocalization. For example, we compute the Manders' Colocalization Coefficients, 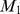 and 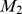, [73]:
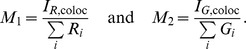



All information computed about the contact site, including the values of 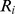, 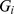, 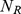, 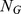, 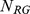, 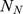, 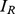, 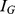, 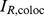, 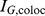, 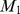 and 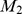 are presented to the user through histograms and written to a spreadsheet as comma separated value (.csv) files. There are a number of other useful utilities in the user interface like Batch Run which helps the user to set up several analyses and process them sequentially as a group.

We validated our methods, both for the spherical approximation as well as localization and the colocalization analysis, by creating uniform spherical shells of known intensities with different sections removed from each channel so that the two channels overlapped in a known way, then compared the results with what we expected. With the parameters noted above, we got identical results for both the total intensities and the Manders' Colocalization Coefficients, showing our methodology reproduced these examples very well.

It should be noted that, while the above description of our analyses includes only two fluorescence channels, the methods are extensible and may also be used to compare three or more fluorescence channels, which will become important as multi-color technology improves. The capability to measure as many color channels as desired, plus membrane stain and DIC, is currently implemented in our analysis tool.

We use relative fluorescence units for receptor quantification. Absolute receptor numbers would be useful, but this requires accurate accounting for background and calibration to standards. We found multiple sources of background in our images, some of which exhibit variable amplitude and spatial heterogeneity in and around contact sites. Additionally, the background in calibration beads is not comparable to the background in our cellular system. These characteristics make accurate background correction for absolute receptor number determination difficult. When averaged over many contacts, comparisons of relative intensity such as we have made are valid.

Our approach was designed to quantify receptor distributions in spherical contact site geometries. The requirement for spherical geometry in our analytical method is fairly lenient because only the contact site surface for analysis is relevant and it must only reside within a spherical shell defined by the chosen minimum (which may be zero) and maximum analysis radii. For instance, our yeasts are not perfect spheres, and we have not experienced difficulty in the analysis of *C. parapsilosis* contacts, even though these yeasts are relatively ellipsoidal (mid-sectional 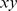 plane major/minor radii ratio = 1.6). Moreover, roughly spherical cellular interaction surfaces are encountered in diverse biological systems where our approach could also be productively applied (e.g., T cell-APC, B cell-T cell, NK-tumor cell, neutrophil attaching to endothelium and neurological synapses). Finally, our method provides several diagnostic plots that describe the radial variation of intensity within the contact sites. These plots assist users to verify and understand results from incompletely spherical systems.
